# ^99m^Tc-MIBI single photon emission computed tomography/computed tomography for the incidental detection of rare parathyroid carcinoma

**DOI:** 10.1097/MD.0000000000012578

**Published:** 2018-10-05

**Authors:** Zejun Chen, Jingjing Fu, Qing Shao, Bin Zhou, Feng Wang

**Affiliations:** a Department of Nuclear Medicine, Nanjing First Hospital, Nanjing Medical University, Nanjing; b Department of Nuclear Medicine; c Department of Thyroid and Breast Surgery, Affiliated Jiangyin Hospital of Southeast University, Jiangyin, Jiangsu, PR China.

**Keywords:** parathyroid carcinoma, primary hyperparathyroidism, PTH, SPECT/CT

## Abstract

This study aimed to evaluate the characteristics of parathyroid carcinoma and to validate the diagnostic value of ^99m^Tc-methoxyisobutylisonitrile (MIBI) single photon emission computed tomography/x-ray computed tomography (SPECT/CT) for differentiating between parathyroid carcinoma and hyperparathyroidism. Four consecutive patients with suspected primary hyperparathyroidism were enrolled in this study and underwent ^99m^Tc-MIBI SPECT/CT, ultrasonography, enhanced CT, and MRI. Serum parathyroid hormone (PTH) and calcium were measured. All primary and recurrent lesions showed high focal uptake on ^99m^Tc-MIBI image, whereas metastatic lymph nodes gave false negative results. The serum PTH was 165.14 ± 90.26 pmol/L, which declined rapidly after surgery. One patient with a persistently high PTH (147.5 pmol/L) after surgery presented with multiple lymphadenopathy in the neck. Higher expression of chromogranin A (CgA) further confirmed parathyroid carcinoma as a rare endocrine tumor. Parathyroid carcinoma is thus usually diagnosed incidentally based on nonspecific multiorgan symptoms of hypercalcemia and hyperparathyroidism. ^99m^Tc-MIBI SPECT/CT may help to localize the parathyroid carcinoma, while MRI is valuable for detecting metastasis. Serum PTH and CgA serve as circulating biomarkers in parathyroid carcinoma, and raised levels of PTH and CgA together with locoregional lymphadenopathy may indicate parathyroid carcinoma. Further studies are needed.

## 1 Introduction

Parathyroid carcinoma is a rare neuroendocrine tumor associated with significantly increased parathyroid hormone (PTH) levels and hyperparathyroidism (HPT). For this reason, the tumor is difficult to distinguish from benign primary HPT and is usually diagnosed incidentally.^[1,2]^ Although several clinical and biochemical features differentiate parathyroid carcinoma from benign primary HPT, the diagnosis and management of parathyroid carcinoma remains difficult for various reasons, including its rarity, a lack of diagnostic tools, and its overlap in symptoms with primary HPT.

There is currently no standardized diagnostic framework for parathyroid carcinoma, and the tumor is usually confirmed by histopathology and characterized by vascular or capsular invasion.^[3]^*En bloc* resection is strongly recommend if parathyroid carcinoma is suspected,^[1,4]^ but fine needle aspiration should be avoided because it cannot differentiate between benign and malignant parathyroid lesions, and is also associated with risks of bleeding and tumor seeding within the biopsy tract.^[4,5]^ Dual-phase ^99m^Tc-methoxyisobutylisonitrile (MIBI) single photon emission computed tomography (SPECT)/x-ray computed tomography (CT), ultrasonography (US), CT, and magnetic resonance imaging (MRI) are recommended before invasive parathyroidectomy to allow precise and accurate localization of the parathyroid lesion.^[1,3]^

In the present study, 4 consecutive patients with parathyroid carcinoma confirmed by histopathological analyses underwent evaluation of biochemical markers and multi-modality imaging, including dual-phase ^99m^Tc-MIBI SPECT/CT, US, enhanced CT, and MRI for the detection and staging of their tumors. The study aimed to evaluate the characteristic of parathyroid carcinoma, and to validate the diagnostic use of ^99m^Tc-MIBI SPECT/CT for differentiating between parathyroid carcinoma and HPT.

## 2 Methods

### 2.1 Patients

Four consecutive patients (2 men and 2 women) with suspected HPT diagnosed between July 2013 and December 2016 were enrolled in the present study (mean age: 53.25 ± 2.63 years, range: 51–57 years). All patients underwent dual-phase ^99m^Tc-MIBI SPECT/CT and US, and enhanced CT or MRI to evaluate the primary lesion and lymphadenopathy. All patients gave their informed consent. The patients’ clinical characteristics are shown in Table 1.

**Table 1:**
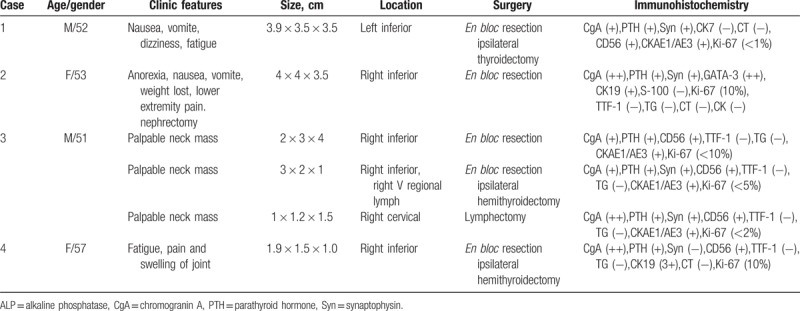
The patient's clinic characteristic.

### 2.2 Imaging protocol

#### 2.2.1 Dual-phase ^99m^Tc-MIBI imaging

Imaging data were acquired using a hybrid SPECT/CT scanner (Symbia T2, 16; Siemens Healthcare, Hoffman Estates, IL). Dual-phase planar images were obtained at 15 and 120 minutes after intravenous administration of 370 to 740 MBq of ^99m^Tc-MIBI using a low-energy, high-resolution, parallel collimator. The images were acquired for 10 minutes in a 128 × 128 matrix with a 20% window centered on the 140-keV photopeak (zoom: 3.2). The SPECT data were obtained with a broad field of view in the neck and mediastinum, as well as dual-head configuration. Thirty-two frames were collected and 16-s projections were acquired with a noncircular orbit step and a 128 ×128 matrix. These SPECT data were then reconstructed using a three-dimensional iterative algorithm. CT acquisition was performed in a similar manner using a tube voltage of 130 kV, 90 to 150 mAs (CARE Dose4D; Siemens Healthcare), a collimation of 6 × 3 mm or 16 × 1.2 mm, and a pitch of 1.2. CT data were reconstructed using a 3-mm slice thickness and a B30 kernel filter (matrix: 512 × 512).

Dual-phase scintigraphy and SPECT/CT data were analyzed using a workstation (e.soft; Syngo Siemens Healthcare) that provided axial, sagittal, and coronal slices of SPECT and CT, and fused images. CT images were used for attenuation correction, tumor localization, and diagnosis. All images were interpreted independently by 3 nuclear-medicine physicians. Images were interpreted in 3 orthogonal planes on the computer monitor of the workstation, and the physicians were unaware of the clinical diagnosis or other imaging examinations.

#### 2.2.2 US and anatomical imaging (CT and MRI)

US was performed using a Philips iU22 scanner (Philips Medical Solutions, Bothell, WA) with a high-resolution (5–12 MHz) real-time scanner. Color Doppler was also performed to assess the vascularity. Longitudinal and transverse images were taken from the clavicles to the mandible, with the patient's neck extended and their shoulders lowered. CT was performed using a Lightspeed 16 (GE Healthcare, Tokyo, Japan) and a dual-source CT scanner (Definite Flash; Siemens Healthcare, Forchheim, Germany). Craniocaudal coverage extended from the skull to the mediastinum, and the images were reconstructed using a 5-mm slice thickness. Helical scans were carried out 60 s after intravenous injection of a contrast medium (ioversol; 80–90 mL; 320 mgI/mL; Tyco, Canada). MRI was performed using a 3-T scanner (Gyroscan ACS-NT; Phillips, Best, the Netherlands) and involved transverse, coronal, and sagittal coverage from the skull to the mediastinum. The scans used a 5-mm slice thickness and included T1-weighted, T2-weighted, and short-tau inversion recovery sequences.

#### 2.2.3 Histopathology and immunoassay

Immunohistochemical staining for chromogranin A (CgA), PTH, and synaptophysin (Syn) was performed to confirm the parathyroid cancer as a neuroendocrine tumor. Specifically, 4-mm sections of formalin-fixed, paraffin-embedded tumor specimens were deparaffinized in xylene and rehydrated in graded alcohols. Endogenous peroxidase activity was blocked by incubating the slides in 3% hydrogen peroxide for 20 minutes at room temperature, followed by rinsing under running water for 5 min. Heat-induced epitope retrieval was carried out in a microwave oven for 30 minutes in preheated, 10 mmol/L citrate buffer (pH 6.0). The slides were transferred to phosphate-buffered saline and then incubated overnight at 48°C in rabbit polyclonal antibodies against CgA (1:50; DaKo), PTH, and Syn, respectively. The next day, the samples were incubated in secondary antibody for 1 hour at room temperature and the substrate chromogen (3.38-diaminobenzidine) enabled visualization of the antigen–antibody complexes via a brown precipitate. Cell nuclei were visualized by counterstaining with hematoxylin-eosin (blue). Omission of the primary antibody provided a negative control. All slides were evaluated independently by 2 investigators who were blinded to the clinical data.

## 3 Results

A total of eight lesions comprising 4 primary lesions, 2 recurring lesions, and 2 cervical lymphadenopathies were detected. The primary tumor size was 3.45 ± 1.03 cm (range: 1.9–4 cm). The mean serum PTH was 165.14 ± 90.26 pmol/L (range: 46–263 pmol/L), which declined rapidly after surgery in 3 patients (mean: 1.38 ± 1.1 pmol/L, range: 0.3–1.5 pmol/L) but remained high in one patient (147.5 pmol/L) who presented with multiple lymphadenopathies in the neck. The mean serum calcium level was 3.32 ± 0.69 mmol/L (range: 2.88–4.42 mmol/L), which fell after surgery (mean: 2.33 ± 0.18 mmol/L, range: 2.17–2.55 mmol/L), and the mean serum ALP was 266.62 ± 202.7 U/L (range: 80.9–559 U/L). The biochemical data are detailed in Table 2.

**Table 2:**
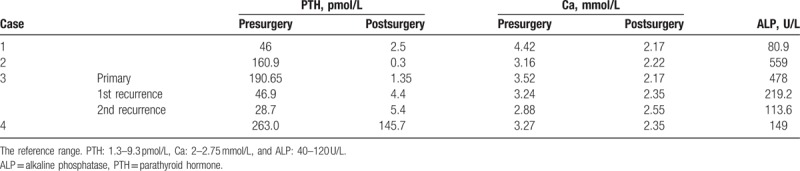
The serum PTH, Ca, and ALP levels of 4 cases of parathyroid carcinoma.

All 4 patients were followed-up for >1 year (range: 14–38 months). Serum PTH levels declined rapidly after surgery in 2 patients, one with a serum PTH level of 1.8 pmol/L and a serum calcium level of 2.4 mmol/L, and the other with a serum PTH level of 2.3 pmol/L and a serum calcium level of 2.2 mmol/L. Multiple pulmonary nodules were detected by CT in one patient with repeated recurrences, suggesting pulmonary metastasis at 38 months (serum PTH: 75.6 pmol/L, calcium: 3.48 mmol/L). The final patient was followed-up for 15 months and presented with multiple lymphadenopathies in the neck, with a serum PTH level of 88 pmol/L and calcium level of 3.17 mmol/L.

### 3.1 Image interpretation

**Figure 1:**
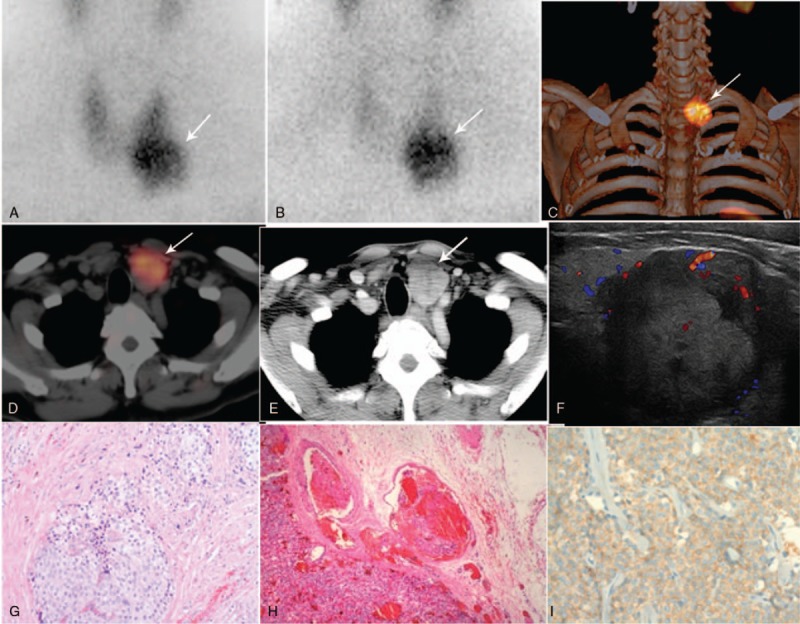
A large, hyper-vascular lesion in the left neck detected by multi-modality imaging in parathyroid carcinoma. (A) Early phase and (B) delayed-phase images of ^99m^Tc-MIBI scintigraphy. (C) Maximum intensity projection fusion image and (D) fusion image. (E) Enhanced CT showing a large hyper-vascular mass with cystic-necrotic degeneration behind the thyroid. (F) A heterogeneous hypoechoic mass with vascular invasion on US. (G, H) Histopathology: hematoxylin-eosin staining (×100). (I) High CgA expression in the tumor (×400). CgA = chromogranin A, MIBI = methoxyisobutylisonitrile, SPECT/CT = hybrid single photon emission computed tomography/x-ray computed tomography, US = ultrasonography.

**Figure 2:**
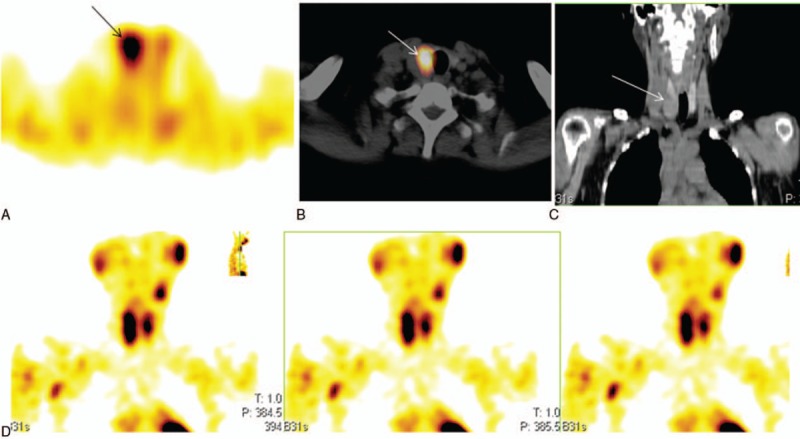
Dual-phase ^99m^Tc-MIBI SPECT/CT showed intense uptake in the right inferior parathyroid. (A) Transaxial image, (B) fusion image, (C) coronal CT, (D) coronal image. MIBI = methoxyisobutylisonitrile, SPECT/CT = hybrid single photon emission computed tomography/x-ray computed tomography.

**Figure 3:**
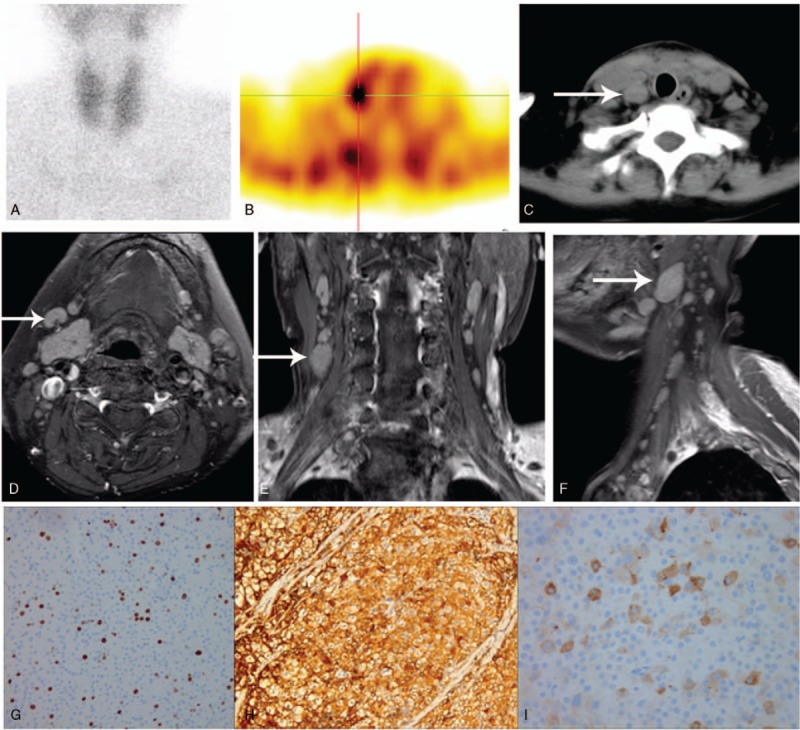
Dual-phase ^99m^Tc-MIBI SPECT/CT, MRI, and immunochemistry confirmed parathyroid carcinoma (A) Planar image showed no significant radiouptake in the parathyroid before surgery. (B) Focal uptake in the lymph node. (C) CT confirmed lymph node involvement. (D–F) Follow-up MRI (6 months after surgery), fat-suppressed, T2-weighted image showing multiple lymphadenopathy. Histopathological and immunohistochemistry. (G) Proliferation index (Ki-67) was 10% (×100), (H) PTH expression was significantly elevated (×400), and (I) CD56 was positive (×400). MIBI = methoxyisobutylisonitrile, PTH = parathyroid hormone, SPECT/CT = hybrid single photon emission computed tomography/x-ray computed tomography.

All primary lesions and 2 recurrent lesions showed high focal uptake on dual-phase ^99m^Tc-MIBI SPECT/CT images. Surgical exploration demonstrated that fusion imaging had identified the precise locations of the lesions (Figs. 1 and 2). However, lymph node metastases produced false negative results, as confirmed by enhanced CT and MRI (Fig. 3). Two primary and one recurring lesion were heterogeneous, hypoechoic masses, with lobulated contours on US. One primary lesion was missed because there were multiple lesions within the thyroid. Three primary and one recurring lesion on CT manifested as enhanced masses behind the thyroid, and one patient with cervical lymphadenopathy on MRI showed enlarged lymph nodes in the bilateral neck.

### 3.2 Histopathology

Immunohistochemistry staining showed significantly increased expression of CgA, PTH, and Syn in 3 patients, suggesting differentiated parathyroid adenocarcinoma, which further confirmed parathyroid carcinoma is a rare endocrine tumor, CgA, PTH, and Syn may served as specific marker of parathyroid carcinoma, serum CgA and PTH may be circulating markers. The immunohistochemical results are shown in Table 1.

## 4 Discussion

Parathyroid carcinoma can occur either sporadically or as part of a genetic syndrome, with an overall incidence of 3.5 to 5.7 per 10 million.^[5]^ In China, 5.96% of primary HPT cases are caused by parathyroid carcinoma, as revealed by serum calcium screening and the widespread use of high-resolution US.^[6]^ However, there may be geographic and ethnic differences in its incidence.^[2,7]^ The etiology of parathyroid carcinoma remains unclear, though a history of radiation exposure to the neck is a known risk factor.^[4]^ In addition, hereditary HPT–jaw tumor syndrome and multiple endocrine neoplasia types 1 and 2A are associated with both benign and malignant parathyroid tumors.^[1,8,9]^ A previous retrospective study demonstrated that parathyroid adenoma and thyroid cancer can be risk factors for parathyroid carcinoma.^[10]^

None of the current patients had any obvious hereditary factors, but suspected parathyroid adenoma was finally confirmed as parathyroid carcinoma. All patients show elevated serum calcium and PTH levels, larger tumor size, and vascular and capsular invasion. We therefore propose that parathyroid carcinoma should be strongly suspected when presurgical examination reveals lymphadenopathy in the surrounding tissues, as well as vascular invasion.

Parathyroid carcinoma tends to occur in the inferior gland, and is usually associated with local invasion and distant metastases. It also frequently recurs within 2–3 years after the initial operation, invading the surrounding tissue and spreading to contiguous structures in the neck.^[5,11,12]^ All the primary lesions in the present study occurred in the inferior gland, and one patient suffered recurrences and pulmonary metastases, while another patient presented with multiple lymphadenopathies in the neck. Dual-phase ^99m^Tc-MIBI scintigraphy and SPECT/CT has shown high sensitivity for the detection of parathyroid adenoma, including ectopic adenoma in the mediastinum, while hybrid SPECT/CT can be used to precisely localize adenomas and is thus valuable for guiding surgery.^[13–15]^ However, ^99m^Tc-MIBI SPECT/CT has not previously been fully validated for the detection of parathyroid carcinoma, though ^99m^Tc-MIBI-avid lesions both in early and delayed images may indicate benign adenoma.^[16]^ In the present study, ^99m^Tc-MIBI SPECT/CT showed all the primary parathyroid carcinoma lesions, but planar imaging missed the primary lesion in case 2, giving a false negative result.

CgA and Syn are specific biomarkers for the identification of neuroendocrine tumors, and were analyzed in the current study. CgA, Ki-67, and Syn were positive in all the primary and metastatic lesions, suggesting that they might be valuable markers for validating the diagnosis of parathyroid carcinoma. Serum CgA may be a particularly valuable circulating tumor marker for early diagnosis. The use of ^68^Ga-labelled somatostatin analogs has been well documented for the detection of neuroendocrine tumors; however, the uses of ^68^Ga-labeled somatostatin analogs and positron emission tomography/CT have not been fully recognized in the diagnosis of parathyroid carcinoma and may warrant further investigation. Similarly, ^18^F-fluorodeoxyglucose positron emission tomography/CT should be employed to detect distant metastases.^[17]^ MRI is a valuable technique for detecting parathyroid adenoma because of its higher resolution, and the combination of ^99m^Tc-MIBI planar imaging and SPECT/CT with MRI may thus be of great value for differentiating between parathyroid carcinoma and benign adenoma. Malignant parathyroid carcinoma should be considered in patients with HPT and locoregional lymphadenopathy. Failure of PTH to decrease significantly after surgery could exclude ectopic parathyroid adenoma, and metastasis secondary to parathyroid carcinoma should thus be suspected, with MRI representing a valuable tool for further diagnosis.

## 5 Conclusion

Parathyroid carcinoma is a rare neuroendocrine malignancy that presents with variable clinical characteristics. ^99m^Tc-MIBI SPECT/CT is valuable for localizing the primary lesion, while serum PTH serves as a robust biomarker of presurgical parathyroid carcinoma and an excellent indicator of recurrence and metastasis. CT and MRI provide complementary modalities to detect recurrence or metastasis. Neuroendocrine tumor biomarkers are also useful for confirming the diagnosis of parathyroid carcinoma.

## Author contributions

**Conceptualization:** Feng Wang, Zejun Chen, Jingjing Fu, Qing Shao, Bin Zhou.

**Data curation:** Feng Wang, Zejun Chen, Jingjing Fu, Qing Shao, Bin Zhou.

**Formal analysis:** Feng Wang, Zejun Chen, Jingjing Fu, Qing Shao, Bin Zhou.

**Investigation:** Zejun Chen.

**Methodology:** Feng Wang.

**Project administration:** Feng Wang, Zejun Chen, Jingjing Fu.

**Resources:** Feng Wang, Zejun Chen.

**Supervision:** Zejun Chen.

**Visualization:** Zejun Chen, Jingjing Fu, Qing Shao, Bin Zhou.

**Writing – original draft:** Feng Wang, Zejun Chen.

**Writing – review & editing:** Feng Wang, Zejun Chen.
